# FMRI Effective Connectivity and TMS Chronometry: Complementary Accounts of Causality in the Visuospatial Judgment Network

**DOI:** 10.1371/journal.pone.0008307

**Published:** 2009-12-14

**Authors:** Tom A. de Graaf, Christianne Jacobs, Alard Roebroeck, Alexander T. Sack

**Affiliations:** 1 Department of Cognitive Neuroscience, Faculty of Psychology and Neuroscience, Maastricht University, Maastricht, The Netherlands; 2 Maastricht Brain Imaging Center, Maastricht, The Netherlands; James Cook University, Australia

## Abstract

**Background:**

While traditionally quite distinct, functional neuroimaging (e.g. functional magnetic resonance imaging: fMRI) and functional interference techniques (e.g. transcranial magnetic stimulation: TMS) increasingly address similar questions of functional brain organization, including connectivity, interactions, and causality in the brain. Time-resolved TMS over multiple brain network nodes can elucidate the relative timings of functional relevance for behavior (“TMS chronometry”), while fMRI functional or effective connectivity (fMRI EC) can map task-specific interactions between brain regions based on the interrelation of measured signals. The current study empirically assessed the relation between these different methods.

**Methodology/Principal Findings:**

One group of 15 participants took part in two experiments: one fMRI EC study, and one TMS chronometry study, both of which used an established cognitive paradigm involving one visuospatial judgment task and one color judgment control task. Granger causality mapping (GCM), a data-driven variant of fMRI EC analysis, revealed a frontal-to-parietal flow of information, from inferior/middle frontal gyrus (MFG) to posterior parietal cortex (PPC). FMRI EC-guided Neuronavigated TMS had behavioral effects when applied to both PPC and to MFG, but the temporal pattern of these effects was similar for both stimulation sites. At first glance, this would seem in contradiction to the fMRI EC results. However, we discuss how TMS chronometry and fMRI EC are conceptually different and show how they can be complementary and mutually constraining, rather than contradictory, on the basis of our data.

**Conclusions/Significance:**

The findings that fMRI EC could successfully localize functionally relevant TMS target regions on the single subject level, and conversely, that TMS confirmed an fMRI EC identified functional network to be behaviorally relevant, have important methodological and theoretical implications. Our results, in combination with data from earlier studies by our group (Sack et al., 2007, Cerebral Cortex), lead to informed speculations on complex brain mechanisms, and TMS disruption thereof, underlying visuospatial judgment. This first in-depth empirical and conceptual comparison of fMRI EC and TMS chronometry thereby shows the complementary insights offered by the two methods.

## Introduction

Cognitive neuroscience today knows several fundamentally different methods to study brain function. These methods may conceptually be divided into functional neuroimaging versus functional interference techniques. *Functional neuroimaging* aims to identify which brain regions are activated during the execution of certain mental functions. Methods such as electro- or magnetoencephalography (MEG or EEG), positron emission tomography (PET), and functional magnetic resonance imaging (fMRI) are all suitable methods to measure brain activity in humans that engage in sensory, motor, or cognitive processing. *Functional interference* techniques actively intervene in neural processing, for instance by permanently or transiently changing (often disrupting) the neural mechanisms at work. Invasive interference techniques, including cooling, microstimulation, and lesioning, are mainly used in animal studies. The only non-invasive interference techniques that can be safely used in human neuroscience are transcranial direct current stimulation (tDCS) and transcranial magnetic stimulation (TMS).

When comparing non-invasive functional neuroimaging methods (e.g. fMRI) and non-invasive functional interference techniques (e.g. TMS), the latter offer the purported advantage that causal inferences can be made: if disruption of neural processing in a brain region results in changed behavioral performance (e.g. longer reaction times for a given task), this brain region is said to be causally involved in, or functionally relevant for, the behavior that is measured. TMS can thus probe causal ‘structure-function relations’ of various regions in a brain network [1]. Through clever experimental design TMS can also be used to investigate connectivity or information flow within functional networks, in at least two ways: 1) by using two TMS coils on different sites in rapid succession, to find the optimal stimulation onset asynchrony that leads to the highest behavioral effect (e.g. along the lines of [2]), or 2) by measuring the optimal time window of TMS stimulation for different brain regions in separate sessions; subsequently comparing these timings to infer which regions were functionally relevant at which time points (e.g. [3], [4]). Such time-resolved TMS has been referred to as TMS chronometry [5].

In recent years, however, functional neuroimaging has moved beyond conventional brain mapping and is now challenging the monopoly on causality analysis ascribed to functional interference methods. For fMRI especially, new analysis techniques are increasingly applied to reveal interactions between brain regions, beyond mere activations in the brain; an approach that has been referred to as the ‘functional integration’ view [6], [7]. These analyses can be subdivided into functional connectivity and effective connectivity. *Functional connectivity* (FC) has been defined as correlation between remote neuro-physiological events in the temporal domain [6]. FMRI can measure FC, for instance, by tracking correlation of BOLD signal fluctuations between areas. By comparing measures of FC during an experimental condition and during rest or a control condition, found connectivity can be shown to be task-specific and therefore interpretatively meaningful (for a recent review of FC analysis in fMRI, see [8]. *Effective connectivity* (EC) has been defined as directed influence from one region to another [6], and can thus be interpreted as causal interregional dynamics. For fMRI, different models have been developed to analyze such influence on the basis of (temporal patterns in) BOLD signals [9]. Most of these models are confirmatory: they require pre-specification of network regions and anatomical connections. Subsequently, various hypothesized models are tested and compared, in terms of directed influences between these regions and along these connections [10].

A recent development in EC analysis is data-driven EC mapping. Granger causality mapping (GCM: [11]) is such a method, which has the advantage of not requiring any pre-specification of anatomical regions involved or hypothesized directions of influence. GCM may therefore be used as an exploratory technique. Granger causality mapping aims to identify, with reference to a seed region Y, which other brain regions (e.g. X) engage in directed interactions with region Y. In other words, GCM determines whether the activity in brain region X “Granger causes” the activity in region Y. The method maps the extent of this Granger causation towards (or vice versa: from) region Y, for all areas in the brain [11], [12]. GCM has successfully been applied to study causal interactions in several recent studies on visuospatial imagery [13], visuospatial judgment [14], motor systems [15], [16], and cognitive set switching [17].

Thus, we have two methods that lay claim to causality analysis and connectivity analysis in the brain; TMS (chronometry) and fMRI EC. But do they really investigate the same processes? The sort of causality analysis involved in fMRI EC seems fundamentally different from the interventional work done with TMS. While fMRI EC (e.g. GCM) ‘passively’ measures the hemodynamic events that occur in the brain, TMS actively disrupts, or modulates, neural processing. As a result, while fMRI EC may be able to measure causal influences *within the brain* (where in GCM ‘causal’ is defined as Granger causal, see [11]), TMS is able to measure causal influences of brain processing *on behavior* (where ‘causal’ is defined as the consequences of active intervention). Also, both fMRI EC and TMS chronometry, in their different ways, can investigate information flow. FMRI EC does this intrinsically, by mapping causal influence between regions. TMS chronometry does this by investigating the relative timing of functional relevance for behavior in different brain regions. Both are claimed to analyze some measure of causality in the brain, and both can be used to investigate network function and network dynamics (i.e. information flows). But since these methods differ fundamentally in terms of dependent variables and mechanisms of measurement, it remains an empirical question how fMRI EC and TMS chronometry relate.

Recent studies have interleaved TMS with fMRI measurements, to estimate the BOLD effects of TMS throughout the brain, effectively providing some middle ground. These early studies have revealed that TMS can have remote effects, throughout anatomical [18]–[20] and task-specific functional [21] brain networks. There are indications that these remote effects can be functionally relevant for behavior [22], [23]. Moreover, our group recently showed these remote effects to be state-dependent: namely cognitive task-specific [21]. In that study on visuospatial judgment, TMS over posterior parietal cortex (PPC) resulted in BOLD signal decreases in PPC and in remote regions including middle frontal gyrus (MFG). Importantly, this TMS-induced right fronto-parietal network effect was only found during TMS over right, but not left, PPC and only during active visuospatial judgment. Moreover, both local and remote task-specific BOLD signal decreases correlated highly with the amount of TMS-induced behavioral impairment on the visuospatial judgment task. This result suggested that the TMS-affected network may have been functionally relevant as a whole. Importantly, this suggestion was supported by a high degree of spatial overlap between the aforementioned network of TMS-induced BOLD signal decreases and an fMRI functional connectivity map that was referenced to right PPC and specific to the visuospatial judgment task [21]. This overlap constituted early evidence for a direct relation between fMRI FC and TMS network effects.

However, several questions remain unresolved and unstudied to this day. First, were the remote effects in right MFG during TMS over right PPC really functionally relevant for behavior? Second, if the remote TMS effects were confined to the functional (visuospatial) network; could fMRI functional or effective connectivity analysis of the visuospatial network reliably reveal and localize these remote regions within MFG in individual participants? Third, generally; how do TMS effects and fMRI connectivity relate? And fourth, specifically; how do the causality and information flow analyses of fMRI EC and TMS chronometry relate?

The current study addressed these questions of interaction, causality, and functional relevance. Since Sack et al. [21] found a close correspondence between the TMS-affected visuospatial network and the fMRI FC visuospatial network, we decided to exploit this correspondence by using the exact same stimuli and tasks in a new combined fMRI and TMS study. Here, we located which part of PPC was specifically activated during visuospatial judgments, using fMRI general linear model analysis. We then used fMRI effective connectivity analysis in reference to this activated PPC cluster in order to exactly localize in every participant which precise region within MFG was functionally and effectively connected to PPC during visuospatial judgments, thus revealing for each participant the fronto-parietal visuospatial judgment network. We subsequently used fMRI EC-guided TMS Neuronavigation for each participant, in order to apply TMS in four experimental time windows over both regions; the task-specifically activated right PPC as well as the task-specifically effectively connected right MFG. This way we could investigate and compare the time course of TMS-induced behavioral effects for PPC and MFG. Our GCM analysis allowed us to investigate the information flow within the brain from a passive measurement, in-brain dynamics perspective. Due to the large conceptual differences between the two methods already mentioned (and expounded in the Discussion below), no direct correspondence could be expected *a priori*. But particularly in light of the common terminology, including ‘causality’, ‘dynamics’, and ‘information flows’, an empirical comparison should enlighten us on the relation between the methods.

Thus, we directly compared the directions of information flow suggested by fMRI EC analysis and the results proposed by TMS chronometry, and here present a discussion of empirical and conceptual similarities and differences between the two methods.

## Methods

### Participants

15 healthy participants (7 males) were tested in this study. Of them, 13 completed the TMS experiment. One participant first started having migraine auras between the fMRI and TMS experiments, and was therefore excluded from TMS measurements. Another participant was excluded because she did not return for the last TMS session. All had normal or corrected-to-normal vision and no history of neuropsychiatric disorders. The experiment was approved by the local medical-ethical committee (“Medisch Ethische Commissie azM/UM”), written informed consent was obtained before participation. Participants were screened for fMRI and TMS experimentation safety by a medical supervisor, and received monetary compensation.

### Stimuli and Task

Participants were presented with visual stimuli on a projection screen inside the MRI scanner, or on a TFT computer monitor. The stimuli consisted of schematized analogue clocks with yellow rims and two either white or yellow hands (13/33 yellow). The hands of the clocks formed different angles, categorized as small or large (13/33 small). Each stimulus was projected for 300 ms at center fixation. Participants were asked to fixate at all times, aided by a grey fixation cross between stimuli. All stimuli and fixation crosses had the same luminance.

**Figure 1:**
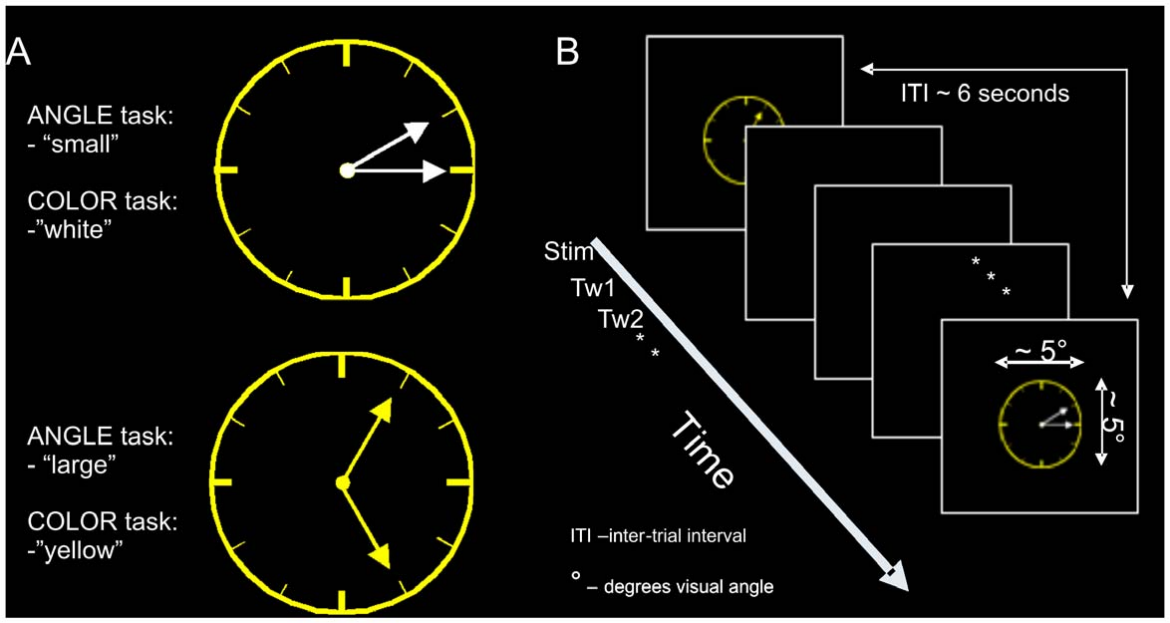
Stimuli, task, and experimental design. A. Two examples of the visual stimuli with task-specific correct responses. B. Illustration of design of both the fMRI and TMS experiments.

There were two tasks: a visuospatial judgment (ANGLE) task and a color judgment (COLOR) control task. In the ANGLE task, a discrimination had to be made concerning the angle between the clock-hands. Participants pressed a right index finger button for clocks with *small angles* (30 or 60 degree angles between the hands) and a right middle finger button for clocks with *large angles* (bigger than 60 degrees). The discrimination thus constituted a visuospatial judgment of angle-size. In the COLOR task, a discrimination had to be made concerning the color of the clock-hands. Participants pressed a right index finger button for clocks with *yellow* hands, and a right middle finger button for clocks with *white* hands. A non-visuospatial judgment of color was thus required, making the COLOR task our control task (see Figure 1 for stimuli, tasks, and design).

Stimuli were presented, and response times recorded, using Presentation software (Neurobehavioral Systems, San Francisco, CA). Response speed and accuracy were equally emphasized in instructions to the participants, although only reaction times were analyzed based on previous work [21].

### FMRI Parameters

The experimental fMRI-design was mixed; blocks of stimuli were presented in a rapid event-related design. Each block contained 11 trials (10 task trials and one null-trial). Throughout a block the task was constant. The task for each block was made known to the participant prior to the block, in the form of a one-letter cue: ‘A’ for ANGLE, ‘C’ for COLOR. The order of blocks was pseudo-randomized, as was the order of trials within the blocks. For the first 10 participants, a total of 28 blocks ( = 280 task trials), divided equally over two fMRI functional runs, were presented (see also [14]). From the last five participants, only a single run (700 volumes) was acquired, which still allowed robust localization of the TMS target regions. Within blocks, the inter-trial interval was jittered around 3000–4500 milliseconds. Time between blocks was 7500 milliseconds, including the 2000-millisecond task instruction.

MRI imaging was performed using a 3 Tesla Siemens Allegra scanner (Siemens, Erlangen, Germany). A standard transmit-receive head coil was used to obtain high-resolution anatomical (ADNI, T1-weighted, Flip Angle (FA)  = 9 degrees, TR = 2250, TE = 2.6 ms, 192 slices, Field of View (FoV)  = 256 mm, isotropic voxel resolution of 1×1×1 mm3) and functional (T2*-weighted echo-planar imaging; FA = 60 degrees, TR = 1500, TE = 28 ms, acquisition gap  = 500 ms, 18 oblique contiguous slices, slice thickness  = 5 mm, FoV = 224 mm, 64×64 voxel matrix, voxel resolution  = 3.5×3.5×5 mm3) images. Participant hearing was protected using ear plugs and headphones. Head movement was restricted using foam pads.

FMRI data were processed using Brainvoyager QX (Brain Innovation, Maastricht, the Netherlands). Pre-processing included interscan slice acquisition time correction, linear trend removal, temporal high-pass filtering to remove low-frequency drifts, and rigid-body transformation of data to the first acquired image to correct for motion. Functional data were coregistered to anatomical data per participant.

For individual right hemispheres, the grey-white matter boundary was determined to segment and reconstruct the cortical surface [24]. Functional data in volume space were projected onto surface vertices in a direction perpendicular to the grey-white matter boundary, and thus converted to surface space. Based on these reconstructed brains, and a reconstructed mesh of an individual participant's head, we could use Brainvoyager TMS Neuronavigation software (Brain Innovation, Maastricht, the Netherlands) to target directly on the individual hemispheres the functionally localized PPC and task-specifically effectively connected MFG for each participant.

### FMRI EC Analysis and TMS Target Localization

For fMRI functional data analysis, BOLD time courses of individual voxels were regressed onto a pre-specified model in a conventional GLM. Model predictors were based on 300 ms events convolved with a hemodynamic response gamma function [25]. Separate predictors were implemented for ANGLE clock presentations, COLOR clock presentations, and INSTRUCTIONS (‘A’ and ‘C’ conjoined in one model predictor, since this predictor served only to decrease error in the general linear model). PPC was localized in each participant as follows: a GLM conjunction analysis of ANGLE vs. baseline and COLOR vs. baseline ((A>B)∧(C>B)) was performed, revealing regions where activity was modulated by both tasks in an individual participant. From these regions, the cluster around PPC with the highest difference in activity between ANGLE and COLOR (ANGLE-specific) was determined. On a RFX group level, we showed that this PPC was significantly more active during ANGLE than during COLOR [14]. This region was thus engaged in visuospatial judgment [21], [26] and served as the starting point for connectivity analysis of the visuospatial network.

Connectivity analysis was performed using Granger causality mapping (GCM: [11]), an exploratory connectivity analysis technique. Per participant, the identified PPC cluster was seeded into GCM analysis to locate MFG. This two-stage procedure was selected prior to measurements, for two reasons. First, this procedure would yield the direction of influence between PPC and MFG on a single-subject basis. Second, previous work had revealed that GCM clusters do not necessarily coincide with GLM activation results. In fact, clusters identified by GCM but not by GLM have been found to be functionally relevant [16].

### TMS Parameters

There were three TMS sessions, involving stimulation of PPC, MFG, or SHAM stimulation. Each session took place on a different day, subsequent sessions separated by at least two days. The order of sessions was counterbalanced across participants. Each session involved 20 blocks of 12 trials each, ten blocks of ANGLE trials and ten blocks of COLOR trials. These blocks were pseudo-randomized within sessions. Between blocks were breaks with durations determined by the participant. Prior to actual measurements, and during breaks, the otherwise dimmed lab room lighting was fully turned on to prevent and reverse dark-adaptation. Participants were given an ear plug for their right ear to protect hearing and to minimize distraction from the auditory stimulation of TMS pulses. They were seated ∼110 cms from a computer screen that displayed the stimuli. The visual angle of the clock stimuli was ∼5 degrees. The TMS conditions involved triple-pulse TMS, with a frequency of 30 Hz. Biphasic pulses were administered by use of a figure-8 coil (MC-B70). The coil handle pointed lateral-posterior, at a 45 degree angle to the midline, for PPC (resulting in lateral-medial, posterior-anterior initial current direction), and pointed lateral-anterior, at a 45 degree angle to the midline, for MFG (resulting in lateral-medial, anterior-posterior initial current direction). Stimuli were presented and TMS pulses were triggered using Presentation software (Neurobehavioral Systems, San Francisco, CA).

On TMS trials, the three pulses were presented in one of four experimental time windows, time-locked to the start of a stimulus (S). The pulses were administered at either (1) 67, 100, 133 ms, (2) 167, 200, 233 ms, (3) 267, 300, 333 ms, or, (4) 367, 400, 433 ms. A time control condition involved TMS pulses applied just after participants responded. Please note that, because participants did not receive TMS pulses prior to their response, this time control condition is in most respects equivalent to a no-TMS condition. Since pulses were administered, however, we will continue to refer to this condition as ‘time control’ (TC). Initially another time window was tested, but since participants in this condition responded early around half of the time, we left this ambiguous time window out of further analyses (responses in other time windows prior to the last TMS pulse were not excluded).

We thus analyzed TW1, TW2, TW3, TW4 as experimental conditions of interest, one time control condition (TC), and SHAM TMS as a sham control condition (SC). The time windows, including TC, were pseudo-randomized across trials, balancing the number of time windows per individual block of 12 trials. To prevent carry-over effects, (pseudo-randomly jittered) inter-trial intervals of 6000, 7000, or 8000 ms were adopted. Stimulation in the TMS conditions was at an intensity of 120% of individual motor threshold.

To ensure stimulation of the functionally localized target sites, we used Brainvoyager TMS Neuronavigation software (Brain Innovation, Maastricht, the Netherlands). Neuronavigation was based on frameless stereotaxic coregistration of the participant's head and the TMS coil by means of ultrasound. Three ultrasound emitters were placed on the participant's head and three on an attachment to the TMS coil. Thus having a plane to represent the head and one to represent the coil, coregistration of these 3-dimensional objects in a single space was achieved: a digitizer pen indicated the locations of predefined landmarks on the skull and on the TMS coil. This procedure allowed us to track in real-time the relative position of head and coil, which made it possible to, online, monitor the brain region stimulated. This procedure has been shown to yield superior methodological and statistical power to detect TMS effects [27], [28]. The coil was held and properly positioned by one experimenter, standing behind the participant.

In the SHAM TMS condition, a sham coil was held and positioned above a site midway between the PPC and MFG target sites. Participants went through the same experimental procedure, including pre-experimental explanations, administrative steps, and neuronavigation setup. They were told that due to low stimulation intensity, peripheral stimulation was unlikely. Several participants believed the session to be genuine and were surprised by our debriefing. Our sham stimulation thus controlled for unspecific effects of TMS stimulation, including the clicking sound of pulse administration, the pressure of a coil on the scalp held by the experiment, and all environmental factors (e.g. the ultrasound emission from the neuronavigation system). Further, additional control was offered by our use of multiple tasks (controlling for task-specificity), sites (controlling for regional specificity), a time window beyond average response times (time control: similar to no-TMS but with the same anticipation of TMS pulses), and multiple time windows (controlling for temporal specificity).

Based on previous research [21], [26] we expected only to find effects of TMS on reaction times (RT) and focused thereon in our analyses. We did confirm that no differences in accuracy occurred between conditions or time windows. Due to a general trend in reaction times over TMS time windows (see Results), we calculated a TMS effect score. We aligned all ANGLE-trials and COLOR-trials per stimulation condition (PPC, MFG, SHAM) per time window, and then subtracted these RTs. The resulting reaction time difference scores (RTdif), or TMS effect scores, were compared between stimulation conditions and time windows, after outlier removal. Outlier removal excluded condition-specific deviants beyond 2.5 standard deviations in RT. Missing values were treated by insertion of scores equal to within-subject and within-condition average, to ensure equal sample sizes across conditions to be compared. Specifically, paired-sample t-tests (one-tailed) were used to statistically compare the RTdif means across different time windows, per stimulation condition, within-subject. Since any TMS-induced differences between the two tasks were conflated into a sensitive single score, the essential control for non-specific TMS effects and general task effects lay in our evaluation of differences between RTdif scores across time windows, between RTdif scores in TMS conditions versus the time-control window, and between RTdif scores in TMS conditions and in the SHAM TMS condition (sham control).

(For this control condition, we collapsed the RTdifs across time windows of the SHAM condition. This was to be conservative: the small fluctuations in RTdif scores across time windows [most likely due to simple error] were in the opposite direction as compared to the TMS-induced RTdif scores. Thus, time-window specific comparisons between TMS RTdifs and SHAM RTdifs would have increased the statistical significance of results in the time windows that already showed significant effects.)

## Results

### FMRI Effective Connectivity in Single Participants

**Figure 2:**
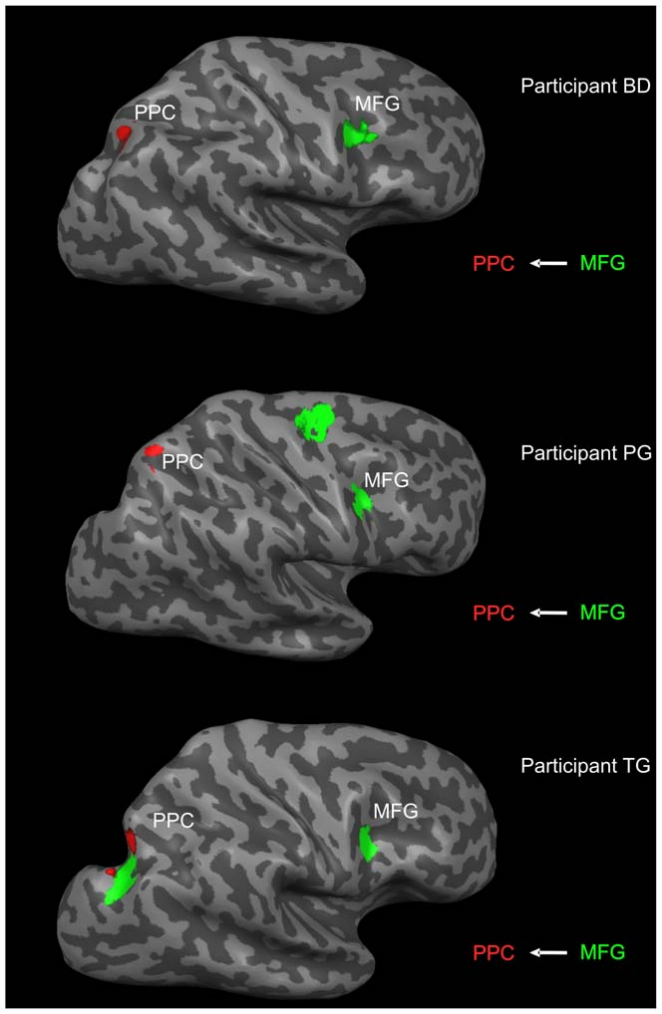
Effective connectivity maps of three participants. Effective connectivity maps in three representative participants are shown on individual, partly inflated, cortical surface reconstructions. Green regions (e.g. MFG) send influence to the red reference region (PPC).

We successfully isolated task-specific PPC clusters in each participant (see Methods). Importantly, seeding these PPC clusters into GCM effective connectivity analysis also clearly, and unambiguously, identified a cluster within MFG for each participant. We previously showed that the MFG cluster was statistically significantly connected to PPC on the RFX group level [14], and here show that effective connectivity analysis for fMRI data during the visuospatial judgment condition could reveal the task-specific effectively connected region within MFG *on an individual subject level*. The direction of influence was invariably frontal-to-parietal, flowing from MFG to PPC. For three participants, the effective connectivity map belonging to the ANGLE condition, in reference to PPC (red), is illustrated in Figure 2 (regions projecting to PPC are shown in green).

**Figure 3:**
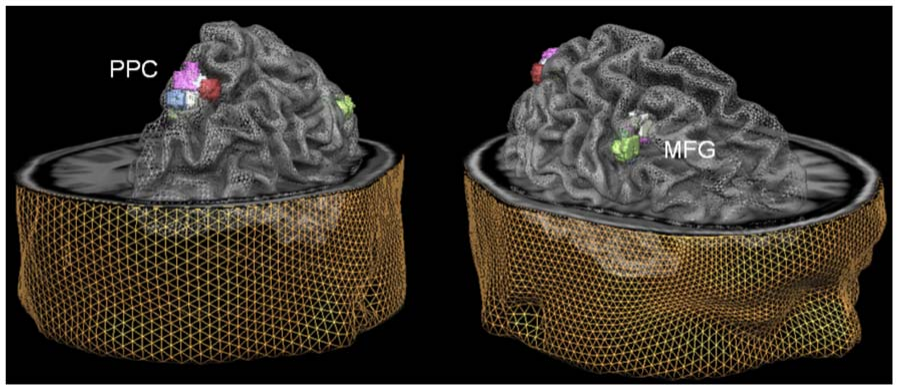
Anatomical location of TMS stimulation sites. For nine individual participants, the TMS targets are shown in colored mesh blobs based on visuospatial task-specific activation (PPC, left) or visuospatial task-specific effective connectivity (MFG, right) clusters. The see-through TMS targets and exemplary head and brain meshes were all transformed to Talairach space for this illustration.

To determine the precise locus of TMS stimulation, we decreased activation or connectivity map thresholds until only the most strongly task-specific voxels (for PPC) or the voxels most strongly effectively connected to PPC (highest GCM values, for MFG) remained. No fixed statistical thresholds were used in this procedure, since the goal here was only to determine the TMS target regions. These were transformed into surface clusters and are displayed for the first nine participants in Figure 3 to illustrate anatomical location and inter-individual spread of target clusters (for this image the surface clusters and exemplary brain were transformed to common Talairach space [29]). Table 1 provides more information on the anatomical locations and spread, by listing the Talairach coördinates for the individual PPC and MFG clusters in the same subjects as shown in Figure 3.

**Table 1:** Talairach coordinates for individual MFG and PPC clusters.

	PPC			MFG		
subject	X	Y	Z	X	Y	Z
1	21	−69	46	37	0	38
2	15	−72	38	33	9	27
3	16	−71	46	44	1	30
4	26	−70	46	47	−1	32
5	18	−73	41	41	4	26
6	25	−75	29	43	−5	36
7	19	−68	58	42	−1	33
8	17	−65	45	45	−2	34
9	26	−71	33	33	1	30
average	20	−70	42	41	1	32
average deviation	3,33	1,96	5,76	3,73	2,47	2,82
standard deviation	4,36	2,92	8,50	5,10	3,97	3,96

The nine PPC and nine MFG clusters shown in Figure 3 are listed here in terms of Talairach coordinates. This allows inspection of inter-individual spread and main loci of TMS stimulation.

### TMS Raw Reaction Times per Condition

**Figure 4:**
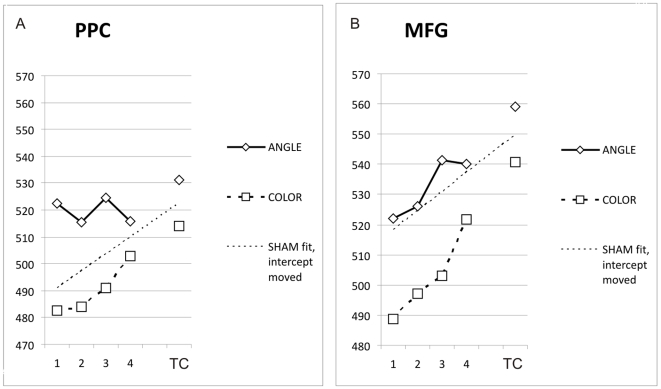
Reaction times during TMS conditions. A. The pattern of average reaction times for all participants (vertical axis, in ms) for the ANGLE condition (solid line) and COLOR condition (dashed line) separately, per time window (horizontal axis), during TMS over PPC. Also shown is a linear fit of the average SHAM data over time windows (with shifted intercept to ease visual comparison of trend, see main text). A clear trend can be discerned in all data, with task- and time window-specific deviations. (TC  =  time control.). B. Same as in A., but during TMS over MFG.

The overall average reaction time for ANGLE was 535.79 milliseconds (ms) (SD = 139.35), the overall average reaction time for COLOR was 512.42 ms (SD = 149.49). Figure 4 displays average reaction times over time windows, for ANGLE and COLOR conditions separately (diamonds and squares, respectively), for TMS over PPC (4A) and TMS over MFG (4B). From these graphs it becomes clear that there was a general trend in reaction times: as TMS came later, in reference to the stimulus onset, reaction times were higher. This trend was clear for all TMS conditions. To confirm that this trend was not due to the neural consequences of TMS administration, we examined the trend in the data from our SHAM TMS condition. The rising linear trend was apparent in SHAM also. We fitted a linear trendline to the average SHAM data (averaged between the two tasks for each time window), and moved the intercept of this trendline so that it fell exactly between the ANGLE and the COLOR TC values. In Figures 4A and 4B, this adapted trendline can be seen, which not only shows how steep the rise in RTs was during SHAM, but also makes it easy to eyeball the differences between the TMS effects on ANGLE and COLOR as compared to the ‘normal’ rising trend.

(The coefficient of the trendline was not changed, of course, only the intercept. The adapted intercepts for PPC and MFG respectively were 484.6 ms and 511.9 ms. The original SHAM trendline intercept averaged for ANGLE and COLOR was 513.9 ms. The trendline coefficient is 6.3228 ms per time window. For the sake of completeness, the trendline formulas for ANGLE and COLOR SHAM data separately were RT = 5.66*TW+525.6 [for ANGLE] and RT = 6.99*TW+502.14 [for COLOR]).

This rising trend of reaction times over time windows can be explained by a general tendency of participants to await the pulses. It was most likely on any given trial that a response could not be made before the TMS pulses (or SHAM clicks) had come, therefore, on trials where the pulses came later some extent of waiting was automatic and a delay resulted. It is because of this general trend that the most sensitive and controlled measure of TMS effect in this study was a difference in RT between ANGLE-trials and COLOR-trials (RTdif). This score was not confounded by any general trend over time, making comparisons of TMS effects between time windows possible.

A few qualitative observations from Figure 4 present themselves. In PPC, the COLOR control trials seemed to follow this trend, whereas ANGLE trials did not, for the first 3 TWs. This suggests that an increase in reaction times might have been induced by TMS on the ANGLE trials for the first three TWs. For TMS over MFG, a rising trend in both ANGLE and COLOR trials can be discerned. However, the trend seems less steep for ANGLE trials, and a relatively large increase in reaction time on ANGLE is apparent in TW 3. This latter observation is particularly apparent in the comparison with the SHAM trendline. To quantify these observations and test them for significance, we calculated RTdif per TW and stimulation site. Whichever *general* trend was in the raw reaction times, it would be similar for ANGLE and COLOR trials, without TMS interference. Therefore, a difference score of reaction times for ANGLE and COLOR trials was calculated (RTdif, see Methods). Any differential effect of TMS on the visuospatial judgment condition (ANGLE) as compared to the control condition (COLOR) should become apparent when subtracting these scores.

### TMS Effect Scores

**Figure 5:**
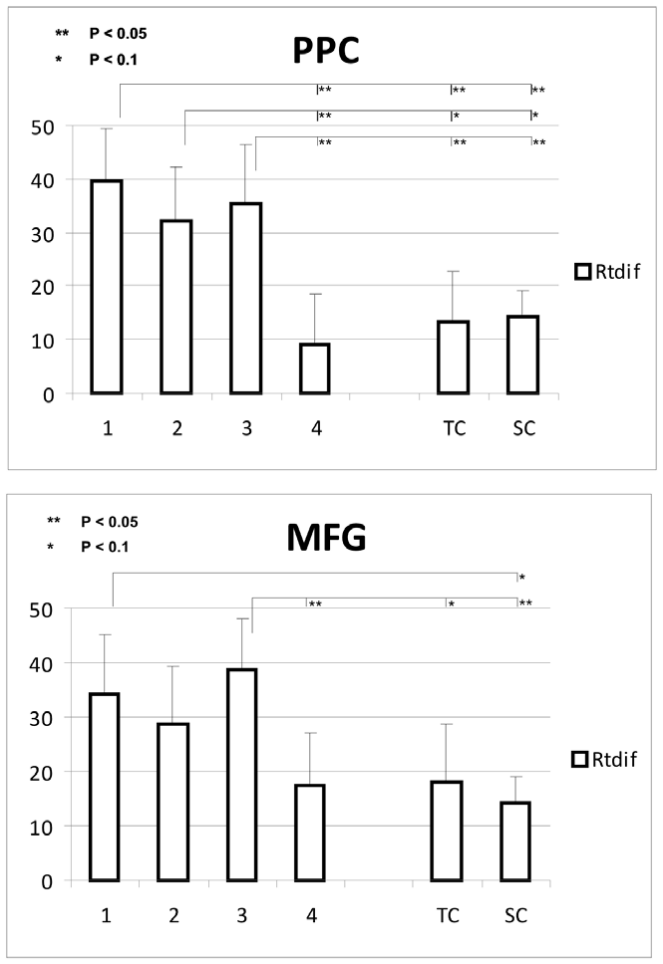
TMS effects over time for stimulation of PPC and MFG. A. The average RTdif scores, or TMS effect scores (vertical axis, in ms, error bars are SEM, RTdif scores are reaction times of ANGLE trials minus reaction times of COLOR trials, see Methods), for all participants, per time window (horizontal axis), for PPC. Also shown by asterisked connections are the results of statistical pairwise comparisons (see methods). (TC  =  time control; SC  =  sham control.). B. Same as in A., but during TMS over MFG.

Figure 5 illustrates the pattern of RTdif scores, or TMS effect scores, over time. Per TW, RTdif is shown in a bar graph for PPC and MFG separately, alongside the RTdif for the time control condition (TC), and the average RTdif for the SHAM TMS control condition (SC). Also illustrated, by asterisked connections, are statistically significant differences and statistical trends in RTdif scores between time windows and control conditions.

For PPC, RTdif in TW1 was significantly higher than both the time control (TC) (t(259) = 2.13; P<0.05) and sham control (SC) (t(259) = 2.18; P<0.05). RTdif in TW2 was marginally higher than both TC (t(259) = 1.42; P<0.1) and SC (t(259) = 1.42; P<0.1). RTdif in TW3 was significantly higher than both TC (t(259) = 1.74) and SC (t(259) = 1.68; P<0.05). RTdif in TW4 was not significantly higher than either TC or SC. These data suggest that TMS had an effect in time windows 1 and 3, and provide some indication that it may have had an effect in time window 2. However, this effect was no longer present in time window 4. In fact, RTdif was significantly higher in all three early time windows when compared to TW4 (TW1-TW4: t(259) = 2.33; P<0.05, TW2-TW4: t(259) = 1.66; P<0.05, TW3-TW4: t(259) = 1.96; P<0.05).

For MFG, RTdif in TW1 was marginally higher than SC (t(269) = 1.63; P<0.1). RTdif in TW2 was not significantly higher than either control condition. RTdif in TW3 was marginally higher than TC (t(269) = 1.57; P<0.1) and significantly higher than SC (t(269) = 2.12; P<0.05). RTdif in TW4 was not significantly higher than either TC or SC. The marginal significance of TW1 vs. SC (P = 0.052), and the marginal significance of TW3 vs. TC (P = 0.059), were nearly statistically significant, approaching the 0.05 threshold. Altogether, these data suggest that TMS had an effect in time window 3 specifically, although there was some indication that there was also an effect in TW1. Either way, the effect was wholly absent by the time of TW4. This was again confirmed statistically: RTdif was significantly higher in TW3 as compared to TW4 (t(269) = 1.74; P<0.05).

Overall, it seems that both TMS over PPC and TMS over MFG had an effect on task-specific reaction times, as measured by RTdif scores. At first glance, the statistics suggest that TMS had an effect when applied over PPC in (TW1, [TW2], TW3), and when applied over MFG in ([TW1], TW3). But we do not think that these results should be taken to indicate differences between PPC and MFG in terms of temporal pattern of functional relevance, for several reasons. First, the overall pattern of results (i.e. mean RTdif scores across time windows) was not dissimilar between stimulation sites. Generally, we found an increase of RTdif in the early time windows (TW1, TW2, TW3), thus until 333 ms after stimulus onset, for both stimulation sites. This increase was unequivocally gone in TW4. Second, the anatomical differences between PPC and MFG could have been responsible for a lower statistical power for MFG. For MFG, we found more variation in the data. We attribute this higher variability of TMS effects to the anatomical differences between the stimulation sites PPC and MFG. The functional cluster MFG, in several participants, was located deeper in the brain, sometimes almost hidden in the middle frontal sulcus, thereby further from the scalp and further from the TMS coil. Previous studies have indicated that the stimulation intensity over motor cortex required to illicit a muscle twitch rapidly increases with distance of coil to cortex [30]–[32]. Thus, in stimulating MFG in different participants, the effect of TMS was dependent on an interaction between the individual cortical excitability and the distance of the MFG cluster from the TMS coil (the depth of the functional cluster). For some participants, this may have led to no, or a lower, effect of TMS in TW1 (and perhaps TW2) over MFG, increasing overall variability. Since PPC was more consistently located on a gyral bank, closer to the scalp, TMS was likely to disrupt cortical processing to a more similar extent across participants. Third, and perhaps most important: to directly address the issue of potential differences in TMS effects between PPC and MFG, we statistically analyzed the differences between the two stimulation sites per time window using paired samples t-tests (two-tailed). RTdif for PPC and MFG were *not* significantly different for any of our time windows: TW1 (t(259) = 0.33; P = 0.74), TW2 (t(259) = −0.19; P = 0.85), TW3 (t(259) = −0.48; P = 0.63), TW4 (t(259) = −0.68; P = 0.50), or time control TC (t(259) = −0.45; P = 0.65). Thus, we conclude that the effects of TMS on PPC and MFG were not significantly different over time, confirming a similar pattern of temporal involvement for both regions, as revealed by TMS.

In summary, TMS can affect task performance both when applied to PPC, and to MFG, up to a time of 333 ms from stimulus onset. By extension, these results indicate that effective connectivity analysis, in reference to a known functionally relevant region (PPC), correctly and accurately delineated a second task-relevant region, namely MFG. Conversely, we could also conclude that the network identified by fMRI EC was behaviorally relevant. Concerning a direct comparison between the two methods' results; as indicated in the Introduction, similar conclusions ought not to be expected *a priori*, since the two methods are so different in underlying logic, dependent variables, and as we will argue below – brain processes measured. In the current study, an *apparent* contradiction arose when comparing the timing information in our fMRI EC and TMS chronometry data. FMRI EC data revealed that information flows from MFG to PPC (see also [14]). TMS chronometry did not reveal differences between the timing of functional relevance of PPC and MFG, thus suggesting that they were functionally relevant at the same time. Taken together, these findings suggest that fMRI EC and TMS chronometry are linked, but not necessarily convergent. This issue will be addressed in more detail below.

## Discussion

The current study addressed several questions of causality and interaction in brain network function. We aimed to reveal functionally relevant TMS target regions with fMRI EC analysis. Using TMS Neuronavigation to stimulate fMRI EC-identified clusters in single participants, we thus tested the behavioral relevance of an fMRI EC functional network underlying visuospatial judgment. Moreover, the time-resolved aspect of our TMS design allowed us to evaluate the commensurability of TMS chronometry and fMRI EC, in terms of suggested information flows. And therefore, to evaluate the extent to which both methods are overlapping, or complementary, in terms of insights yielded.

We were able to show that fMRI EC analysis could indeed successfully define, in individual participants, precise target regions for TMS. Specifically, we showed that regions (MFG) that were task-specifically effectively connected to known functionally relevant brain areas (PPC) were themselves also functionally relevant for behavior. This has implications in three respects: 1) in our study the fMRI-revealed effective connectivity network was behaviorally relevant, in the sense that disruptions of revealed network nodes had a causal effect on behavior (which is something that can only be determined empirically by combined TMS and fMRI EC studies such as the current study) – this is important for fMRI EC research, 2) upon further confirmation (e.g. [16]) fMRI EC might become a useful tool to identify TMS target regions – this is important for TMS research, 3) we could provide indirect evidence for functional relevance of remote neural effects of TMS found by Sack et al. [21]. As explained in detail above, in a recent study our group could reveal that parietal TMS had similar BOLD effects in local PPC and remote MFG. These effects overlapped spatially with GCM functional connectivity analysis referenced to the stimulated PPC. We here used the same tasks and stimuli. On the assumption that our localization methods identified the same PPC and MFG, our data show that MFG is a functionally relevant region, which by extension suggests that the parietal TMS-induced remote BOLD effects in MFG revealed by Sack et al. [21] may also have been functionally relevant. Therefore, this study provides indirect evidence (constituted by our offline TMS over MFG results) for the functional relevance of remote neural effects of TMS (the decrease of MFG activation found by Sack et al. [21] during TMS over PPC). We speculate below on mechanisms of brain network function, and TMS disruption thereof, on the basis of our combined studies.

This study also addressed the comparability of TMS chronometry and fMRI effective connectivity analysis, the latter exemplified by Granger causality mapping [11]. Effective connectivity analysis on PPC identified a frontal region MFG in each individual participant that drove the activity in PPC along a frontal-to-parietal direction of influence. Targeting this seeded PPC and the connected MFG with TMS in distinct time windows resulted in time-specific behavioral effects for both brain regions. The TMS results did not confirm the direction of influence as suggested by the effective connectivity analysis. We will now discuss the commensurability of these different types of data (fMRI effective connectivity analysis and TMS chronometry) both conceptually and empirically in more depth, specifically in the context of our results.

### TMS Chronometry versus Granger Causality Mapping in the Current Study

GCM indicated a frontal-to-parietal information flow; revealing an MFG→PPC direction of influence. This would seem to predict functional relevance of MFG before PPC, in the TMS data. However, statistical tests indicated functional relevance of PPC in early time windows (TW1), while no convincing statistical evidence was found for relevance of MFG in these time windows. Also, PPC and MFG ceased to be functionally relevant in the same time window. These are two separate findings that both do not corroborate a MFG→PPC flow of influence. But several caveats ought to be made.

Concerning the first finding; the evidence that PPC was functionally relevant prior to MFG on the basis of statistical tests may have been due to statistical power differences. As explained in the Results section, further statistical testing and inspection of the general pattern of RTdif scores over time led to the conclusion that TMS had similar effects on PPC and MFG over time. Importantly, this finding is still not corroborated by the fMRI EC result of MFG→PPC directional influence.

Concerning the second finding; the disruption of processing in MFG and PPC in TW3 was clear from the data, as was the finding that all TMS effects had ended by the time of TW4. Thus, we may state that PPC was not functionally relevant *after* MFG, as our fMRI EC data would seem to predict. In this light we should point out that the effective temporal resolution of our TMS protocol was 100 ms, since this was the time between the first pulse of the triplet in one time window, and the first pulse in a following time window. Finer-grained temporal processes could not be distinguished. One might therefore argue that – within time window 3 – MFG may have been functionally relevant before PPC, as indicated by GCM. However, we emphasize that our fMRI EC analysis almost certainly could not have picked up on such a temporally fine-scaled process. It has been shown previously that with the current fMRI EC analysis (GCM) and fMRI parameters (TR = 1500 ms), it is highly unlikely that a neural process occurring in under 100 ms would be revealed [11].

To summarize, out of these two intriguing possibilities; 1) fMRI EC analysis has a temporal resolution below 100 ms, the MFG→PPC information flow occurs in between 267 and 367 ms, and the apparent contradiction between fMRI EC and TMS chronometry was due to our design, or 2) TMS chronometry and fMRI EC measured different aspects of brain function, which means that they were complementary rather than competitive; the first option is implausible and the second remains. It thus becomes important to address on the conceptual level the relation between fMRI effective connectivity analysis and TMS chronometry, with respect to their information on causality.

### On the Commensurability of TMS Causality and fMRI Effective Connectivity

*Prima facie*, GCM and TMS chronometry are both measures of causality and information flow. Yet, our data do not support their equivalence in the current setting. There are several conceptual differences between the two methods that are rarely discussed, but argue *a priori* against a *necessary* convergence of resulting data. In the Introduction already, we pointed out one of the most important conceptual differences: TMS investigates the relevance of a brain region *for behavior*, whereas fMRI EC investigates the relevance of a brain region *for the activity in other brain regions*. ‘Causality’ in TMS pertains to behavioral causality, ‘causality’ in GCM pertains to within-brain causality (in the predictive ‘Granger’ sense; [11]). When it comes to information flow, things are slightly more complex. GCM claims to identify the directed influence of one region on another, which to us seems very similar to what ‘information flow’ refers to in common language (although GCM has the problem that it cannot discern whether an influence is direct or indirect: [11]). TMS chronometry as applied here, on the other hand, can only find that one region is functionally relevant before or after another region. Strictly speaking, this only says that both regions are relevant to the task and at different times. There is no measure of information actually flowing between the two regions. In summary, there are arguments for and against the use of ‘causality’ and ‘information flow’ in reference to TMS (chronometry) or fMRI EC. We do not wish to settle any such dispute. We would only urge researchers to be clear and unambiguous in their use and meanings of the terminology applied. What we *do* wish to emphasize, is the various distinctions between the two methods, and the implications for cognitive neuroscience. Let us therefore discuss a few more differences between the two methods.

One important, seemingly obvious difference is the dependent variables of both methods. FMRI EC, as most fMRI methods, relies on hemodynamic (blood flow, blood volume and deoxy-hemoglobin concentration) information. An assumption lies between neuronal firing and hemodynamic responses [see e.g. 33], removing fMRI EC information by one step from the timing of the actual brain activity. Moreover, hemodynamic responses are sluggish; therefore GCM results may reflect processes that follow or precede the actual task performance, which in our case lasted only several hundreds of milliseconds, within trials of several seconds. In-between task-related bouts of visuospatial processing, the neuronal brain was doing other things, such as anticipating the upcoming stimulus, or reflecting on performance on the previous trial. These latter processes would have been stimulus-locked as well, and may have been task-specific. Therefore, the fMRI EC results could reflect information flows inherent to these processes. Though a general problem in fMRI, these factors play no role in time-resolved TMS.

Conversely, TMS has the disadvantage that it is not a passive measurement. In contrast to fMRI, TMS works directly on the neurons [see e.g. 34]. But this also means that TMS alters the very brain function it is studying, perhaps akin to the quantum uncertainty principle in physics. For instance, compensatory processes have been shown to take place in response to TMS [35]. Thus, for example in our experiment, MFG might have been functionally involved in a relevant way in early time windows. Our behavioral data would not reflect this early functional relevance, if other regions compensated for the TMS disturbance, if the role of MFG became increasingly important over time, or if the MFG-related processing became less distributed and more focal over time. Any of these scenarios would remain obscure in a TMS study, but might be observed in an fMRI EC study.

These methodological matters, combined with our conceptual distinctions and caveats on what ‘kinds’ of causality and information flow are measured by fMRI EC and TMS chronometry, *a priori* did not make it necessary for fMRI EC and TMS chronometry results to overlap. Rather, it was an open empirical question whether fMRI EC and TMS chronometry would converge in terms of timing information. In this experiment we could not find evidence that they do. But please note that this does not falsify either of the two methods, nor does it mean that interpretation becomes impossible in the event of an apparent contradiction. After all, interestingly, fMRI EC did successfully map a behaviorally relevant brain network, as indicated by the TMS-revealed functional relevance of PPC and MFG. Therefore, the two methods *are* related and ostensibly have something to offer both when in unison *and* in discord. This suggests that fMRI EC and TMS may be complementary and mutually constraining, and may lead to valuable insights in functional brain organization if used in sensible combination. We will use the visuospatial judgment network as an example. Note that what follows is in large part speculative, and proposed as a hypothesis rather than fixed conclusions. We do think this example provides a comprehensible, and plausible, illustration of what we mean by complementary insights from fMRI EC and TMS chronometry.

### Visuospatial Judgment: Recurrent Connections Suggested by Diverging Methods

Influence followed the frontal-to-parietal direction (suggested by GCM data), but PPC was functionally relevant at the same time as MFG (based on TMS data). In such a situation, TMS data can provide constraints on GCM data and vice versa. If MFG→PPC communication were unidirectional it would seem unlikely that the receiving region (PPC) was functionally relevant at the same time as the sending region (MFG). The alternative is that MFG→PPC information flow was not unidirectional, but merely stronger than the coexisting, but less dominant, PPC→MFG information flow. This possibility of asymmetric bidirectional interaction has been discussed theoretically [11], confirmed empirically in a different setting [36], and could not be distinguished on the basis of GCM data alone. The added information from our TMS data, however, plus data found in previous research (see below), suggests that the interactions between PPC and MFG are bidirectional.

Recurrent connections come in at least two forms. First, a back-and-forth option: a quick feedforward sweep, followed by a feedback sweep [2], [37]. In the case of a back-and-forth loop; information flow from PPC to MFG may have escaped the scope of GCM, which only ‘picked up on’ the slower and more elaborate (frontal-to-parietal) feedback loop. The second option involves continuous reiterative dynamic loops of bidirectional interaction between PPC and MFG (e.g. dynamic re-entry: [38]). In this scenario, although information exchange was continuous and bidirectional throughout task performance, the MFG→PPC influence was simply stronger (see also [36]). Both the quick back-and-forth and continuous reiterative versions of bidirectionality would be, in principle, compatible with our TMS results. We believe, however, that the continuous reiterative form is more plausible in light of previous work, which we now turn to.

BOLD has been shown to reflect input of activity, or local cortical processing, rather than output [39]. Therefore, BOLD effects of TMS might reflect induced changes in input, rather than direct TMS-induced changes of firing rate. Sack et al. [21] revealed that TMS over right PPC resulted in BOLD signal decreases in both right PPC and MFG. We now propose that the neural effects in PPC, due to TMS over PPC, were indirect and due to recurrent processing: *the (TMS-)disturbed output of PPC disrupts input to MFG, which again disrupts input to PPC in turn, and so on*. A TMS disturbance thus may *reverberate* through the integrated network that is currently operating. This suggestion is compatible with evidence from simultaneous TMS and EEG measurements showing that neural effects of a TMS pulse spread further from the stimulation site during wakefulness than sleep [40]. A recent fMRI effective connectivity study by our group also provided evidence for recurrent loops within several nodes of a visuospatial network, including PPC and MFG [14]. In the case of the visuospatial judgment network, bidirectionality would explain the deactivation results found by Sack *et al.* [21], but fast, dynamic, reiterative loops between the two regions would moreover explain the highly similar relation between BOLD and behavioral impairment for both PPC and MFG. The correlation between BOLD signal and behavioral impairment was 0.91 for PPC, and 0.89 for MFG [21]: remarkably high and remarkably similar. We find it difficult to see how such high concordance could be achieved through one back-and-forth loop between the two regions. Continuous reiterative interactions, with a dominant MFG→PPC component, would explain our GCM data and the high correlation data from Sack et al. [21]. This model would also predict that the frontoparietal network as a whole is responsible for visuospatial judgment, and that TMS over either node (PPC or MFG) would disturb the network as a whole and have behavioural effects, and that these effects would likely be found in similar time windows. Our TMS data confirm both predictions.

To summarize, a dominant frontal-to-parietal (MFG→PPC) information flow was suggested by GCM. TMS chronometry and incorporation of previous work [14], [21] constrained these interactions to be bidirectional and possibly continuously recurrent. Although speculative, this interpretation of results serves as an example of how fMRI EC and TMS chronometry findings may be complementary rather than contradictory.

### Conclusion

In the current study we revealed a fronto-parietal information flow in each out of 15 participants using fMRI EC (GCM). Time-resolved TMS over these parietal (PPC) and frontal (MFG) regions showed that both were functionally relevant for visuospatial processing, an important finding particularly in light of previous simultaneous TMS/fMRI work [21]. Moreover, the TMS data were not in clear agreement with the fMRI EC analysis, although the latter analysis did successfully identify functionally relevant TMS target regions (which conversely means that fMRI EC analysis successfully mapped a behaviorally relevant network, underlying visuospatial judgment). We discussed why the two methods may be complementary rather than conceptually overlapping. Furthermore, we used this and previous visuospatial judgment studies as an example to show how these methods can make mutually constraining contributions to the understanding of network function. This resulted in a model of functionally relevant recurrent loops in the fronto-parietal network underlying visuospatial judgment.
